# A Comparison Between the StaRRsed Auto-Compact Erythrocyte Sedimentation Rate Instrument and the Westergren Method

**DOI:** 10.4021/jocmr476w

**Published:** 2010-12-11

**Authors:** Juha Horsti, Riikka Rontu, Auni Collings

**Affiliations:** a Tampere University Hospital, Centre for Laboratory Medicine, Department of Clinical Chemistry, Tampere, Finland

## Abstract

**Background:**

The Westergren method is the golden standard for measuring erythrocyte sedimentation rate (ESR). All ESR methods should agree with the standardized method of the International Council for Standardization in Hematology (ICSH). Citrate samples are commonly used for ESR. This extra sample adds costs and can be inconvenient for the patient. Therefore, some new automated ESR analyzers use EDTA samples, which are available for other hematology measurements.

**Methods:**

We compared ESR measurements with StaRRsed Auto-Compact instrument to the ICSH standardized Westergren method in 200 patient samples.

**Results:**

The correlation between methods was fairly good (R^2^ = 0.72, y = 1.066x 0.24). However, with ESR results over 11 mm/h there were 55 subjects with a difference of over 30% between methods.

**Conclusions:**

This may have led to different treatment suggestions in 25 cases according to age- and gender-dependent normal values. The difference may be caused by two different anticoagulants used, different measuring times and the correlation equation used. The StaRRsed ESR method should be in better agreement with the Westergren method, which is the golden standard. ESR results have notable impact on patient diagnosis and follow-up.

**Keywords:**

ESR; Erythrocyte sedimentation rate; StaRRsed; Westergren method

## Introduction

Estimation of the erythrocyte sedimentation rate (ESR) has a long history dating back to ancient Greece [1] and it has remained one of the most popular clinical laboratory tests to date. The ESR increases, for example, in various infectious diseases, infarctions, malignancies and autoimmune diseases reflecting both the plasma (namely, acute-phase proteins) and cellular properties [namely, the red blood cell (RBC) concentration, RBC surface charge and aggregation] [2-5]. The slow changes in ESR differ from the rapid changes in C-reactive protein and these two analyses complete each other in diagnosing infection, inflammation and in follow-up. ESR is a particularly sensitive indicator of silent and chronic inflammation that is the underlying process in many diseases, for example, in atherosclerosis [6, 7].

The ESR measurement is inexpensive and easy to use in clinical laboratories of various sizes. The methodology and measuring principles vary markedly according to method although, in principle, all methods should be evaluated in comparison with the reference standardized method to obtain harmonization [8]. Also, diagnosis and treatment suggestions should be based on the same age-related normal values [9, 10]. Although EDTA samples would be usable for both ESR and hematology measurements, 1:5 citrate diluted samples are widely used for ESR analysis. The International Council for Standardization in Hematology (ICSH) has prepared recommendations for the measurement of ESR [11] and Thomas et al have given suggestions for the calibration and validation of ESR tests from EDTA samples (ICSH reference method) to the Westergren level [12]. The calibration of ESR is very important for accurate measurements because of the differences in blood sample quality (citrate or EDTA, sampling tubes), measuring principles and measuring times.

Traditional manual methods mostly use the sedimentation principle in the original Westergren pipette or vacuum tube to measure the ESR as the distance that the column of blood cells falls in one hour [8, 13]. These methods widely use citrate diluted samples (4 vol. blood plus 1 vol. citrate). This adds costs in sampling and laboratory logistics and could be inconvenient for the patient due to extra sample taking. Increased sample volumes in clinical laboratories have led to the development of automated ESR analyzers. Some of the automated methods use EDTA as an anticoagulant, which is ideal because of its usability in other hematological measurements. One of the automated systems is Alifax TEST 1 (SIRE Analytical Systems, Udine, Italy), which uses undiluted EDTA samples or diluted citrate samples, rotation force, and photometrical analysis to measure ESR. The method needs a specific gain (calibration) for Westergren method comparison depending on the anticoagulant used. Preliminary results of this analyzer showed a good correlation with the Westergren method [14]. Later, Hardeman et al made a practical evaluation and comparison between the TEST 1 instrument and StaRRsed Auto-Compact (Mechatronics, Zwaag, the Netherlands) using a large material of 680 samples and a newer software version of TEST 1. The correlation between these two methods was again good (R^2^ = 0.90) but further statistical analysis showed that, depending on the instrument that was used, in 11.5 % of the samples the results could lead to different treatment suggestions [15]. Some (semi)automated analyzers, like the Sedimatic 100 (Analys Instrument AB, Sweden), use the original measuring principle and measure the sedimentation of erythrocytes in a vacuum sample tube with citrate buffer as the anticoagulant. The correlation between citrate and EDTA ESR with this instrument was shown to be good (R^2^ = 0.93) [16]. AlFadhli et al also used citrate samples to compare SEDIsystem^TM^ (Becton Dickinson, Vacutainer Systems, USA) with the conventional Westergren method and concluded that a correction factor is recommended because the SEDIsystem^TM^ underestimates ESR [17]. A comparative study between the classic Westergren method and the sealed vacuum extraction method was done by Wiwanitkit et al and it showed very good correlation between the methods (R = 0.99) with a coefficient of variation (CV) below 3% [18]. Moreover, it was also observed that ESR estimation can be made from an EDTA sample without clinically significant differences from the Sedimatic 100 routine citrate method [16].

Our aim in the present study was to compare the 'classic Westergren' method and the StaRRsed method by analyzing the same patient EDTA sample (n = 200) by both measuring principles and also to evaluate the possible diagnosis based on both ESR results. The classic Westergren method results were obtained using the ICSH standardized method [11] and a calibration equation by Thomas et al (R = 0.996) was used to obtain the classic Westergren ESR results in mm/h [12].

## Materials and Methods

### Blood samples

Venous blood samples were obtained from 200 routine hospital patients from whom the ESR was requested. Six samples were collected from a healthy volunteer for the intra-assay CV measurements. The inter-assay CV% in StaRRsed was measured using a commercial SEDRite Plus control (R&D Systems, France). The control was analyzed once daily during a one-month time period. Blood (3.0 ml) was drawn into EDTA tubes (Greiner Labortechnik GmbH, Vacuette cat. no. 454246 or Terumo, Venoject, cat. no.VF-053SDK42) containing K_2_EDTA (1.5 mg/ml). The sample needle (Terumo, Venoject needle, Quick Fit, cat. no. MN-2138MQ) that was used was 0.8 × 40 mm. Samples were mixed well at the time of venipuncture and again just before analysis. Each sample was analyzed first with the StaRRsed analyzer and immediately afterwards with the classic Westergren pipette (Vacuette, Greiner bio-one). All measurements were commenced within 6 hours of blood collection. All procedures were approved by our institution's responsible committee in accordance with the Helsinki Declaration of 1975.

### StaRRsed Auto-Compact instrument

StaRRsed (Mechatronics Manufacturing BV, The Netherlands) analysis is based on the Westergren sedimentation technique although the method is slightly modified. Routinely, 3 ml of K_2_-EDTA-blood is taken for the ESR determination. The instrument uses a vacuum pump to aspirate 1.6 ml of this sample and dilutes it with 0.4 ml of 3.8 % (105 mM) Na_3_-Citrate solution. The diluted sample is then aspirated to the Westergren pipette and the sedimentation is measured using the optical density at 950 nm after exactly 30 min. A correlation curve is then used to transform the results into 60 min measurement time. Finally, results are given in mm/h at 18^o^C using the temperature correction equation in the instrument according to the manufacturer.

### Westergren method

K_2_-EDTA anticoagulated blood samples were mixed for 10 minutes before the test. Samples were aspirated into the Westergren pipette and the distance that the column of blood fell in 1 h was recorded according the ICSH standardized method [4]. The results were changed to original 'Westergren units' using the calibration equation by Thomas et al (y = 0.1072 * x^1.4195^ + 2.1812) (R = 0.996) [12].

### Statistics

The Microsoft Excel 5.0 program and Analyse-it (Analyse-it Software, Ltd, UK) were used for method comparisons. A correlation coefficient, Bland-Altman plot and Passing-Pablok analysis were used in the regression analysis. Accuracy was established by using the 95% confidence interval (CI) for the mean difference between methods. We also evaluated the clinical agreement between the methods. Evaluation of diagnosis was based on the age-dependent normal values by Bottiger [9] and Zauber [10].

## Results

The study included 118 women with the mean age of 61 years and 82 men with the mean age of 64 years. Six samples were collected from a healthy volunteer for the intra-assay CV measurements. The intra-assay CV% was 13.0 % (n = 6) in the classic Westergren method (mean 6.0 mm/h) and 0.0% (n = 6) in StaRRsed method (mean 5.0 mm/h). The inter-assay CV% was measured by using a commercial SEDRite Plus control (R&D Systems, France) and it was 6.1% (n = 28) in StaRRsed.

**Figure 1.**
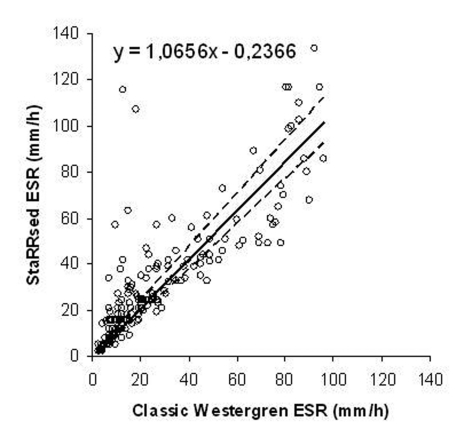
StaRRsed ESR vs. classic Westergren ESR in mm/h according to Passing-Pablok regression analysis (n = 200).

**Figure 2.**
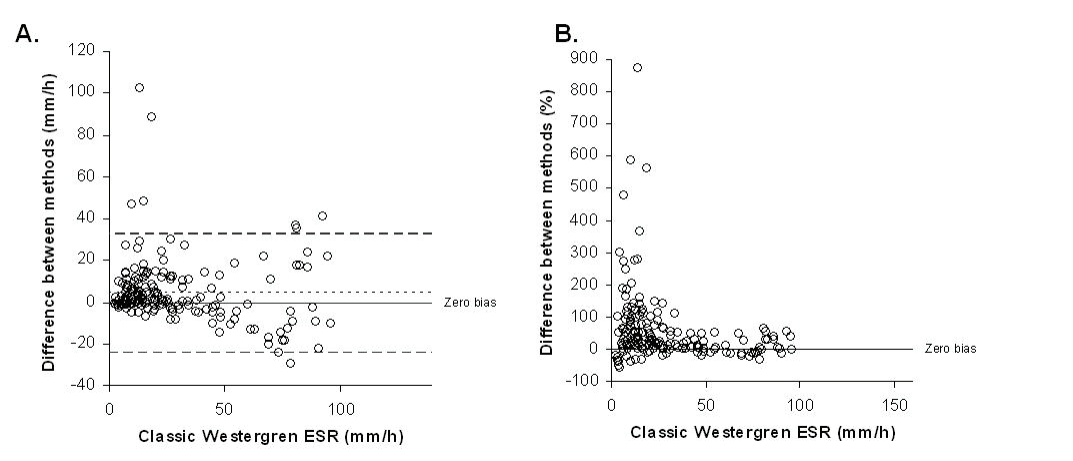
Difference between methods according to classic Westergren ESR results (A) in mm/h, and (B) in percentages. In the panel A, the dotted line indicates 95% limits of agreement (lower -24, 95% CI: -28 to -21; upper 33, 95% CI: 29 to 36).

The mean ESR was 29.99 mm/h in StaRRsed analyzer and 25.80 mm/h in the classic Westergren method. The difference between the averages was 4.1 mm/h (16.2%). The overall correlation coefficient was 0.72 according to the Passing-Pablok method comparison (y = 1.066 x 0.24, 95% CI: intercept -1.137 to 0.966 and slope 0.980 to 1.166). There was a non-linear relationship between the two methods (P value < 0.01) as shown in Figure 1. The difference between methods versus the classic Westergren method both in mm/h and in percentages is shown in Figure 2.

Statistically significant differences were found between ESR values in some samples. With the ESR results over 11 mm/h, 55 samples, namely 27.5%, showed a difference of more than 30%. According to normal ESR values [9, 10], in 25 patients (12.5%) the results of ESR determination could lead to different clinical decisions, depending on which instrument was used. Of these samples, 24 samples showed increased ESR on StaRRsed while only one had increased ESR on the classic Westergren method.

## Discussion

One of the oldest clinical laboratory methods and one that has not been changed over the years is the Westergren ESR procedure. Commercial interests and growing sample numbers have, however, led to the development of high throughput analyzers also in ESR analytics. One of the newer techniques is the StaRRsed Auto-Compact analyzer which is capable of analyzing 135 samples per hour if the 30 min mode is used. This is, of course, an attractive alternative to the older methods that require a lot of hands on time and manual work.

The manufacturer of StaRRsed states that the analyzer fully applies to the recommended Westergren method [11]. It is, however, slightly surprising that blood is first drawn into a primary anticoagulant (EDTA) tube followed by dilution of 4+1 with sodium citrate. This principle deviates from the classic Westergren method clearly. We prefer the ICSH standardized method and correlation according to the calibration equation measured by Thomas et al to get classic Westergren results in mm/h. The method is more reliable as a reference method for ESR measurements for comparisons because only one anticoagulant is used [12]. Also, the normal ESR values for patient diagnosis are based on the classic Westergren method and variations in the sedimentation process might lead to wrong clinical decisions in patient care. The use of two anticoagulants might to some extent lead to differences between StaRRsed and the classic Westergren method observed in the present study: 24 out of 25 clinically relevant samples had a higher ESR in StaRRsed compared to the classic Westergren.

The red cell sedimentation is influenced by a number of interacting factors. Principally, it is due to the difference in density between red blood cells and plasma [19]. An important factor influencing this is erythrocyte aggregation or rouleaux formation. Other factors that have an influence are ratio of red cells to plasma, and changes in erythrocyte surface charge, size, shape, deformability and density [19]. The three-stage sedimentation reaction of erythrocytes through plasma contains: firstly, the red cell rouleaux formation (10 min) in which erythrocytes aggregate in a specific manner; secondly, sedimentation (40 min); and thirdly, cell packing (10 min) which depends both on hematocrit and ESR [20]. The sedimentation time on StaRRsed is user-selectable: if the 30 min measuring time is used, a correlation equation changes the measurements into 60 min results. This leads to the fact that measurements taken at times other than 60 minutes are not directly comparable with those obtained under ICSH recommended conditions. The 30 min sedimentation time may be too short for some samples and could cause errors, especially at the higher ESR levels, even if the average correlation is acceptable. The compensated 30 minute sedimentation obtained with StaRRsed could lead to overestimation of the 60 minute ESR. Kallner studied the temporal development of ESR in vacuum tubes and found that 3% of the samples sediment fast and reach the end of reaction much earlier than 60 minutes [21]. For these patients, using the 30 min reaction time and a correlation equation, the ESR will be highly overestimated. This could, in fact, explain why we obtained higher ESR results in StaRRsed compared to the classic Westergren method. Despite this fact, however, we wanted to perform the comparison in conditions that would be routinely used in ESR analytics to obtain a realistic view of the results.

StaRRsed measures the ESR in ambient temperature, namely within range 18-25^o^C, followed by an automatic temperature correction. Results are thus given at 18^o^C. The temperature correction calculations can vary day-to-day depending on the room temperature and this could cause differences in the patient result levels. However, temperature correction is widely used in many semi-automated and automated ESR analyzers, so the StaRRsed is in that way analogous with other analyzers.

Without question, StaRRsed has many advantages: it offers consumables savings, safety and fluent workflow. One also has to bear in mind when comparing the StaRRsed results with the classic Westergren results, that the manual reading of the erythrocyte sedimentation from the Westergren pipettes is much more prone to imprecision than automatic reading on the StaRRsed, especially when the plasma to cell interface is hazy. There were some outliers in our results that were included into the analysis although the differences could be due to the imprecision in manual reading. This could, in some cases, explain the differences between the two methods that were observed in the present study. Unfortunately, the 3 ml sample volume is only sufficient to measure ESR once in both methods. Duplicate measurements would have been more reliable to confirm or refute the differences that were seen in some samples.

The diversity of factors involved in the ESR often renders the interpretation of sedimentation rates difficult. Since ESR results are indicative in nature rather than being precise and accurate measurements of a specific analysis, it can be considered that differences between results obtained by testing under ICSH conditions and from compensated measurements taken after a shorter sedimentation time are acceptable and clinically insignificant. The differences between StaRRsed and classic Westergren method that were observed in the present study are, in many cases, acceptable and clinically insignificant. However, in long term patient follow-up, the greater than 30% difference observed in some patient results could be misleading in patient care and diagnosis. On the other hand, the automated ESR methods offer significant improvement in report turn-around time and in this way improve the service of the laboratory. StaRRsed has many excellent technical properties but the complexity of the method is increased by the use of two anticoagulants, which affect on RBC surface charge and correlation equations both in reaction time and temperature measurements. To simplify and to improve the quality of the results, it would be better to use undiluted EDTA samples, 60 min measurement time, and a calibration equation by Thomas et al. Since the ESR has a marked role globally in the diagnosis and follow-up in patient care, the different ESR methods should better agree with each other because the reference ranges used are the same.
